# Fabrication of ZnO/Carbon Quantum Dots Composite Sensor for Detecting NO Gas

**DOI:** 10.3390/s20174961

**Published:** 2020-09-02

**Authors:** Ziyang Yu, Liang Zhang, Xiangyue Wang, Dong He, Hui Suo, Chun Zhao

**Affiliations:** 1 State Key Laboratory of Integrated Optoelectronics, College of Electronic Science and Engineering, Jilin University, Changchun 130118, China; yuziyang1991@163.com (Z.Y.); zhangliang18@mails.jlu.edu.cn (L.Z.); hedong@jlu.edu.cn (D.H.); suohui@jlu.edu.cn (H.S.); 2 College of Chemistry, Jilin University, Changchun 130118, China; xiangyue18@mails.jlu.edu.cn

**Keywords:** carbon quantum dots, ZnO, gas sensors, NO

## Abstract

ZnO and carbon quantum dots (CQDs) were synthesized by a hydrothermal method, and CQDs were doped into ZnO by a grinding method to fabricate a ZnO/CQDs composite. The X-ray diffraction and the scanning electron microscope revealed that the as-prepared ZnO has a structure of wurtzite hexagonal ZnO and a morphology of a flower-like microsphere which can provide more surface areas to adsorbed gases. The ZnO/CQDs composite has a higher gas sensitivity response to NO gas than ZnO microspheres. A gas sensitivity test of the ZnO/CQDs composite showed that the sensor had a high NO response (238 for 100 ppm NO) and NO selectivity. The detection limit of the ZnO/CQDs composite to NO was 100 ppb and the response and recovery times were 34 and 36 s, respectively. The active functional group provided by CQDs has a significant effect on NO gas sensitivity, and the gas sensitivity mechanism of the ZnO/CQDs composite is discussed.

## 1. Introduction

Nitric oxide (NO), as a harmful gas that threatens human survival, is widely produced by the combustion of fossil fuels. NO is colorless and odorless, which could damage nerve function, as well as cause neurodegeneration and other diseases at high concentrations [1]. NO is also the simplest biologically active molecule in the body and has played a vital biological role in the cardiovascular, cerebrovascular, immune, neurological, urinary and reproductive systems [2,3,4,5]. Therefore, rapid and accurate detection of NO has very important significance in environmental protection and medical science, and research work related to NO sensors has become active [6,7].

Some detection techniques for NO have been researched such as electrochemical sensing by semiconductor materials. As a wide-band-gap semiconductor sensitive material, ZnO has the advantage of low cost, high sensitivity, simplicity in fabrication and compatibility with modern electronic devices [8]. However, metal oxide gas-sensing materials also have the disadvantages of high operating temperature and low selectivity, which restricts the application of metal oxide materials in gas sensing [9]. Many previous works have demonstrated that the most important factor determining the performance of a gas sensor is the design and synthesis of sensitive materials [10]. Therefore, the preparation of gas-sensitive materials with high sensitivity, good selectivity and low operating temperature is of great significance for the development and application of sensors. Many researchers have designed ZnO in combination with other materials to improve the performance of gas-sensitive materials [11,12].

It seems that CQDs (carbon quantum dots) materials have become an excellent choice to improve the sensitivity performance of materials. CQDs are nanomaterials composed of carbonaceous skeletons which usually contain sp2 hybrid graphite carbon or sp2 sp3 hybrid carbon mixed with amorphous carbon, with a particle size of less than 10 nm and surface groups [13]. CQDs also show fast photo-generated electrons transfer capabilities, superior up-conversion luminescence capabilities and fluorescence characteristics, and they have been successfully used as excellent electron acceptors and donors [14,15,16]. Based on different CQDs precursors, materials with different surface functional groups can be obtained and these functional groups can selectively react with certain gases.

Herein, we have successfully fabricated a ZnO/CQDs composite sensor for electrochemically detecting NO. We firstly synthesized ZnO with a flower-like structure by a hydrothermal method, and then incorporated carbon quantum dots by mechanical grinding and mixing, which avoided the surface functional groups of CQDs from being destroyed during high-temperature reactions. The morphology, crystalline structure and the composition of ZnO/CQDs were characterized and the gas sensitivity of the composite has been investigated in detail. The ZnO/CQDs materials possess excellent NO selectivity, fast response/recovery and, compared with the ZnO without CQDs, the sensitivity properties of the ZnO/CQDs composite were greatly improved, where the results showed that the introduction of CQDs could improve the composite’s response to NO gas. In addition, the sensitive mechanism of the ZnO/CQDs composite has been discussed.

## 2. Experiment

### 2.1. Synthesis of ZnO/CQDs Composite

The ZnO microspheres were synthesized by a hydrothermal route. In a typical synthetic route, glycine (130 mg), Na_2_SO_4_•10H_2_O (130 mg) and Zn(CH_3_COO)_2_•2H_2_O (146 mg), were dissolved in a solvent which was mixed with 4 mL of deionized water and 5 mL of ethanol. After 130 mg of NaOH was slowly added, the solution was magnetically stirred for 30 min to get a white gel which was transferred into a Teflon-lined autoclave (20 mL) and heated at 180 °C for 8 h. The centrifugation, cleaning and drying were performed, and the dried precipitate was calcined at 400 °C for 2 h. The collected product was white powder, and subsequent material characterization was performed on this powder unless otherwise specified. The synthesis procedure of CQDs is described as follows. Four hundred mg L-ascorbic acid, 4 mL glycol and 6 mL deionized water were mixed with a magnetic blender. Then, the precursor was transferred to a 20 mL Teflon-lined stainless-steel autoclave and heated at 160 °C for 70 min without any further passivation [17]. After this bottom-up method for preparing CQDs, the obtained 9 mL CQDs dispersion liquid contained 1.5 g of CQDs. The reaction product CQDs dispersion liquid was cooled down to room temperature, which can be used directly [18]. Define 10 uL CQDs solution (1.67 mg CQDs) doping into 100 mg ZnO powder as 1eq(equivalent). In order to retain the properties of CQDs, the ZnO/CQDs composite was prepared by an appropriate method. After 100 mg of prepared ZnO powder was mechanically ground for 30 min, we then dripped 50 uL of deionized water and 1 eq (2 eq, 5 eq) of CQDs and continued grinding for 20 min, obtaining the ZnO/CQDs (1 eq, 2 eq, 5 eq) composite. Then, we added 50–100 μL deionized water as a dispersant to the prepared ZnO/CQDs (1 eq, 2 eq, 5 eq) composite, and then used a brush with a diameter of 3 mm to evenly coat the dispersed composite on a ceramic tube with gold electrodes. The coated ceramic tube was cured at 150 °C for 1 h, and then a nickel-chromium resistance wire was inserted into the ceramic tube. We welded the ceramic tube and resistance wire on the hexagonal base and covered it with a dust-proof net to obtain a sensor device.

### 2.2. Measurement of the Gas Sensor

**Figure 1:**
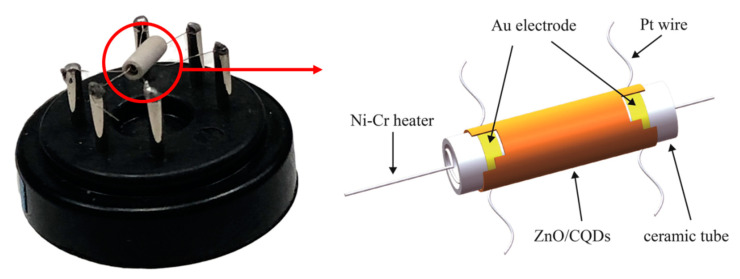
Schematic diagram of device structure.

The gas-sensitive performance was tested by a laboratory-made gas-sensitive test system, which includes gas-sensitive components, gas cylinders, Fluke 8846A digital multimeter, GPD3303S DC power supply and a computer workstation. The working temperature of the sensor is controlled by the nickel-chromium alloy resistance wire in the ceramic tube device. The diagram of the ceramic tube device is shown in Figure 1. The gas-sensitive performance was tested at a temperature of 25 ± 2 °C, and a relative humidity of 25 ± 5%RH. In the test, the sensor was placed in a closed container with a volume of 2.5 L, and the inlet and outlet of the target gas and pure air were controlled through the intake and exhaust valves. The gas response of the ZnO/CQDs sensor can be defined as S = Ra/Rg, however, in an oxidizing atmosphere, gas response S = Rg/Ra. In the formula, Ra and Rg are the electrical resistance of the component in air and the target gas. We define the response and recovery time as the time taken to reach 90% of the final stable resistance change value [19]. Each sample with a different doping ratio was fabricated and tested 3 times or more.

### 2.3. Characterization

An X-ray diffractometer (XRD, Rigaku D/Max 2550, operated at 40 KV/200 mA with Cu-Kα radiation (λ = 1.5406 Å) and a transmission electron microscope (TEM, JEOL JEM-3010, JEOL, Ltd., Tokyo, Japan) were used to determine the phases, crystallinities and chemical compositions of the ZnO/CQDs composite. The microstructure and morphology of the ZnO/CQDs composite was characterized by field emission electron microscopy (FESEM, JEOL, JSM-7500 F, JEOL, Ltd., Tokyo, Japan). The thermogravimetric analysis (TGA) was performed on a Perkin-Elmer TG-7, in an air atmosphere, and the heating rate was 20 K/min.

## 3. Results and Discussion

### 3.1. Material Characterization

**Figure 2:**
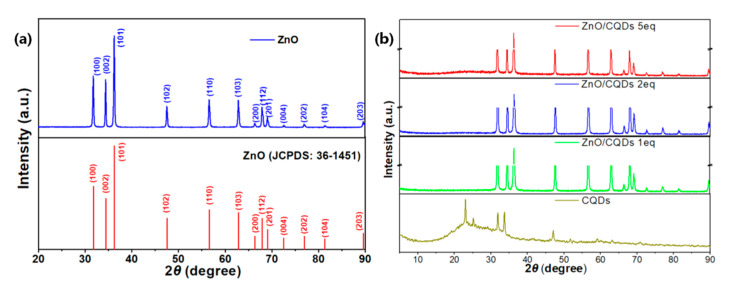
XRD pattern of (**a**) the as-prepared ZnO microspheres; (**b**) the carbon quantum dots (CQDs) and the ZnO/CQDs 1eq (2 eq, 5 eq) composite.

Figure 2a shows the powder XRD pattern of ZnO microspheres. As can be seen from the figure, the diffractogram of ZnO microspheres shows distinct characteristic peaks at 31.7°, 34.4°and 36.2° corresponding to the (100), (002) and (101) planes. Obviously, compared with the standard peak values of ZnO (JCPDS No.36-1451) [20], the ZnO microspheres are a wurtzite hexagonal structure ZnO with lattice constants a = b = 3.243 Å and c = 5.195 Å. Figure 2b displays the XRD pattern of the as-synthesized CQDs and the ZnO/CQDs composite with different ratios of CQDs. The diffractogram of CQDs shows many broad diffraction peaks and sharp diffraction peaks. Broad diffraction peaks of 2θ = 20–30° usually correspond to amorphous carbon and organic material [21], and the sharp diffraction peaks indicate that the material is not ordinary carbon nanoparticles but carbon quantum dots with better crystallinity [22]. Thus, broad diffraction peaks of 2θ = 20–30° can be observed in the XRD diffraction patterns of the ZnO/CQDs composite samples with different CQDs doping ratios, in which the broad diffraction peaks will be more obvious with the increase in the CQDs doping ratio.

**Figure 3:**
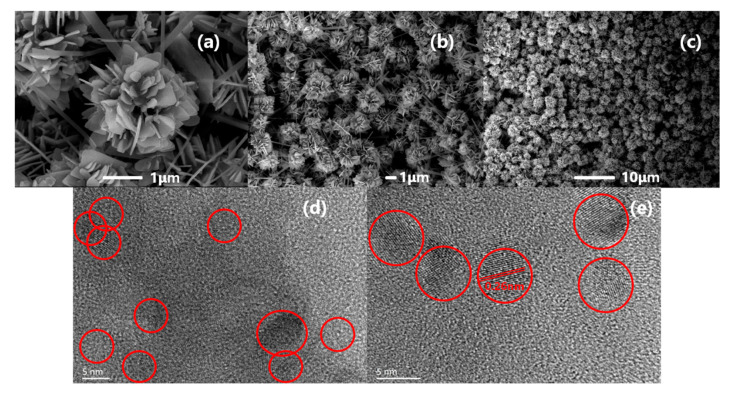
(**a**–**c**) Representative SEM images of the ZnO microspheres; (**d**,**e**) TEM images of the CQDs.

The surface morphology and structure of pure ZnO microspheres were studied by FESEM, and the results are shown in Figure 3a–c. As can be seen from Figure 3a, zinc oxide microspheres show a unique flower-like morphology, and each of them has a uniform size and a diameter of 2~3 μm. Multiple ZnO flakes are stacked on each other to form a hollow sphere. There are crosses between the flakes, but there are little shrouds in each flake of the ZnO microspheres. The thickness of the single flakes can be calculated by the Debye–Scherrer equation [23].

D = Kλ/(βcosθ)

D is the grain diameter obtained by the formula, corresponding to the response intensity of the XRD patterns (Figure 2a) at 31.8°, 34.4°and 36.3° which belong to the (100), (002) and (101) planes. D is also the thickness of the flakes. When we take β as the full width of half maxima (FWHM), the Debye–Scherrer constant K should be 0.89. The λ is 1.5406 Å according to the Cu-Kα radiation of the X-ray diffractometer and the θ is the Bragg diffraction angle. The thicknesses calculated by the Debye–Scherrer equation are 34.1, 40.8 and 33.3 nm. In our previous work, we found that ZnO flakes will thicken as the calcination temperature and precursor hydrothermal time increase [24,25]. For the purpose of maintaining the porous morphology of the ZnO/CQDs composite after doping with carbon quantum dots, we designed smaller and thinner synthesized ZnO microspheres in this work. Figure 3d,e represent the TEM images of the prepared CQDs. TEM samples with a particle size less than 10 nm are required to be stabilized by ultra-thin carbon support films. In order to distinguish CQDs from the carbon background, CQDs can be identified by lattice fringes. The prepared CQDs are carbon nanoparticles with a particle size of about 5 nm, and the lattice fringes are clearly visible, indicating that CQDs have high crystallinity, consistent with the characterization results in Figure 2b. The lattice spacing of the prepared carbon quantum dots is 0.26nm, which belongs to the lattice spacing range of carbon quantum dots (0.20~0.35 nm) [13].

**Figure 4:**
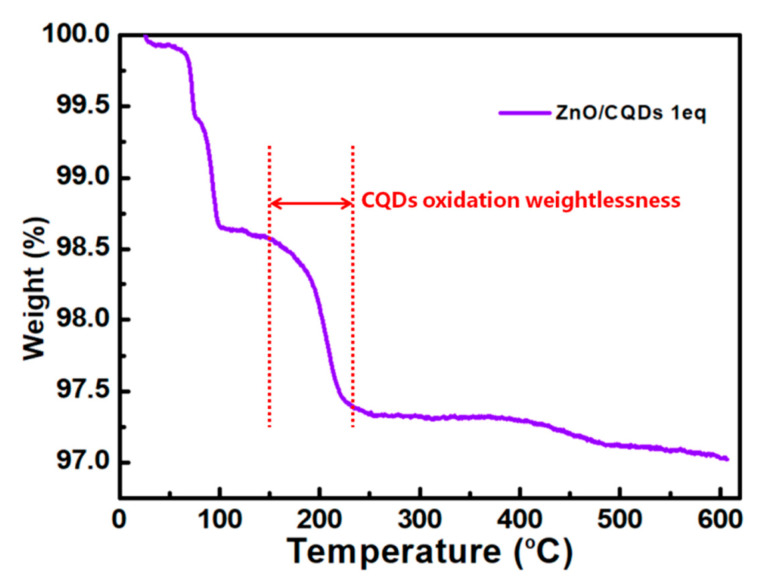
TGA of the ZnO/CQDs 1eq composite.

TGA (thermogravimetric analysis) was determined as the characterization of the ZnO/CQDs material composition. Figure 4 shows the TGA of the ZnO/CQDs 1eq composite, in which the weight loss of 0–100 °C is attributable to the H_2_O adsorbed on the surface of the composite, while the weight loss of 150–230 °C is attributed to the oxidation process of CQDs in the composite material, and it is worth noting that pure ZnO does not have this period of weight loss [26] and there is no obvious weight loss up to 600 °C. It can be known from TGA that the ZnO/CQDs composite contains doped CQDs, but CQDs do not have excellent thermal stability, which is one of the reasons why we choose the preparation method of grinding doping at room temperature.

### 3.2. Gas-Sensing Characteristics

In order to test the gas-sensing performance of the ZnO/CQDs composite material in this work, a ZnO/CQDs composite with different doping ratios (1 eq, 2 eq, 5 eq) was fabricated and the composite was prepared into the sensor device as shown in Figure 1, and the gas-sensing performance test was carried out through the gas-sensitive test system. It should be pointed out that the environmental humidity during the test has a great influence on the gas sensitivity of the material. Excessive humidity will reduce the adsorption of the target gas on the gas-sensitive material, and therefore reduce the gas-sensitive performance of the device [27,28]. Thence, the test was conducted at a relatively constant ambient temperature (25 ± 2 ℃), and a relative humidity (25 ± 5%RH).

**Figure 5:**
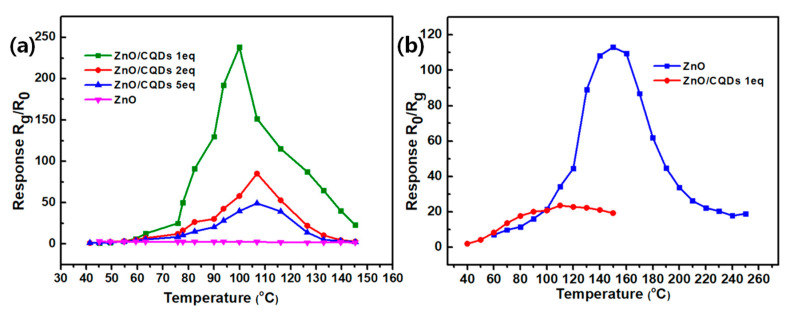
(**a**) Gas-sensing response of ZnO and ZnO/CQDs 1 eq, 2 eq, 5 eq composite at different operating temperatures to 100 ppm NO; (**b**) gas-sensing response of ZnO and ZnO/CQDs 1eq composite at different operating temperatures to 100 ppm H_2_S.

The gas sensitivity of the fabricated sensitive materials with different doping ratios (ZnO, ZnO/CQDs 1 eq, 2 eq, 5 eq) to 100 ppm NO at different operating temperatures is shown in Figure 5a, where the operating temperature range of the sensitive materials was set to 40~145 °C since the CQDs have predictable thermal stability at temperatures below 150 ℃. As exhibited in Figure 5a, among these sensitive materials, ZnO/CQDs 1 eq has the greatest response to 100 ppm NO at an operating temperature of 100 °C, which is 238. For the pure ZnO microspheres, their response to 100 ppm NO was 2.8 at 95 °C, much lower than ZnO doped with CQDs. For other doping ratios such as ZnO/CQDs 2 eq and 5 eq, the maximum responses were 85.2 and 49.2 at 105 °C, respectively, which were also relatively lower compared with ZnO/CQDs 1 eq. The incorporation of CQDs improves the selective adsorption and reaction capacity for NO, due to the active functional groups such as –OH on the surface of carbon quantum dots. The specific sensitive reaction will be explained in the mechanism section. The reason for the lower response caused by the higher CQDs doping ratios is that the high ratio of doping CQDs greatly increases the static resistance of the composite, and this effect on resistance exceeds the resistance change in the presence of NO. An interesting phenomenon is that in our previous work [25], ZnO microspheres have been proven to possess enhanced H_2_S sensitivity. Although the static resistance of ZnO microspheres doped with carbon quantum dots was increased (actually beneficial to the response of H_2_S), the sensitivity of the composite material to H_2_S was greatly reduced (Figure 5b), suggesting that the incorporation of CQDs affects the surface adsorption of chemisorbed oxygen and H_2_S.

**Figure 6:**
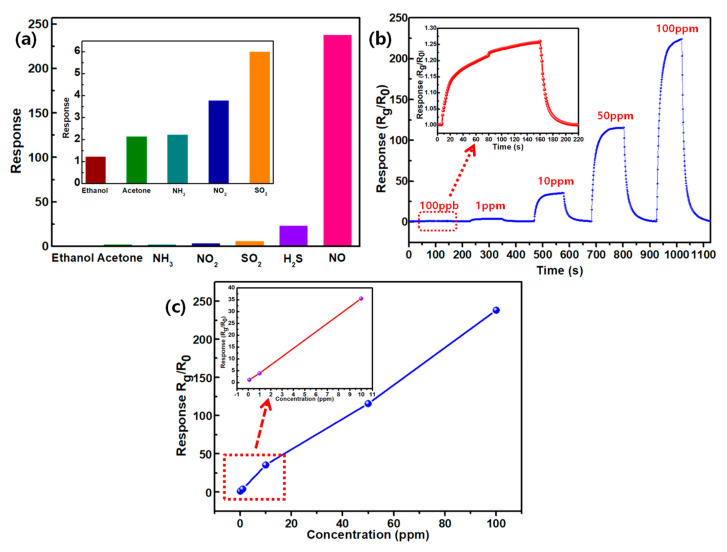
(**a**) The selectivity of the ZnO/CQDs 1eq composite to 100 ppm different gases; (**b**) the dynamic response of the ZnO/CQDs 1eq composite to 0.1−100 ppm NO at 100 °C; (**c**) the response of the ZnO/CQDs 1eq composite to 0.1−100 ppm NO at 100 °C.

The ZnO/CQDs composite also displayed excellent selectivity to NO gas. As shown in Figure 6a, it exhibits the response of the most effective response of the ZnO/CQDs composite with a doping ratio of 1 eq to the gases of ethanol, acetone, NH_3_, NO_2_, SO_2_ and H_2_S. Obviously, compared with NO gas, the ZnO/CQDs composite does not possess a good response to these other gases, and this property could also be attributed to the special reaction of the active functional groups of carbon quantum dots to NO gas. Figure 6b shows the dynamic response of the ZnO/CQDs 1eq composite to concentrations ranges from 100 ppb to 100 ppm of NO at an operating temperature of 100 °C. As the concentration increases, the material shows greater response, and at the same time, as low as 100 ppb, the composite also has a certain response. There is an excellent linear relationship between the response and the concentration of the target gas at low concentrations (Figure 6c), which provides a possibility for the quantitative detection of NO gas.

**Figure 7:**
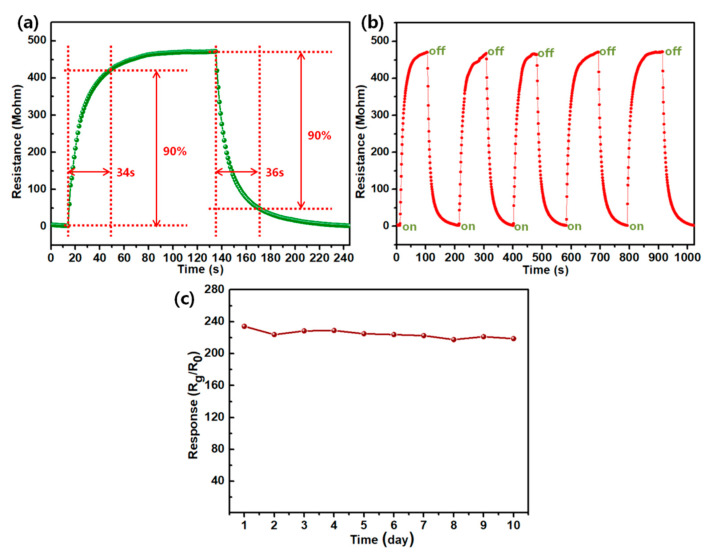
(**a**) The response and recovery of the ZnO/CQDs 1 eq composite to 100 ppm NO; (**b**) the repeatability of the ZnO/CQDs 1 eq composite at 100 °C to 100 ppm NO; (**c**) the long-term stability of the ZnO/CQDs 1 eq composite to 100 ppm NO.

Response and recovery time is an important basis for evaluating whether the sensor is exceptional or not. Figure 7a displays the response/recovery curve of the ZnO/CQDs 1 eq composite to 100 ppm NO at 100 °C. In general, the response and recovery time is usually defined as the time taken to reach 90% of the final stable resistance change value [19], as depicted in Figure 7a, the response and recovery times of the ZnO/CQDs composite can be calculated as 34 and 36 s, respectively. The rapid response/recovery indicates that a reversible surface reaction occurs during the entire gas-sensing reaction process. In Figure 7b, five intake and exhaust gas processes are tested, and the dynamic response curve of the ZnO/CQDs composite to 100 ppm NO at 100 °C was obtained. The dynamic response curves show excellent consistency, which indicates that the device has good stability and repeatability. The long-term stability of the device is also tested (Figure 7c). After 10 days of a long-term stability test, the response of the device decreased slightly. This may be due to the extremely small particle size of the carbon quantum dots, which are easily oxidized during repeated heating and cooling during the test, damaging the structure and properties of the carbon quantum dots [13].

### 3.3. Gas-Sensing Mechanism

As is known, ZnO is an n-type semiconductor. The classic explanation of the sensitive mechanism of semiconductor gas sensors is as follows: In semiconductors, under the excitation of external energy, free electrons transit to the conduction band and become carriers. For n-type semiconductors, the carriers are electrons, and the number of carriers, that is, free electrons, affects the resistance of the semiconductor. The semiconductor sensitive material is suitable for a classic electron depletion layer mechanism [29,30]: The sensitive material was placed in the air, and a certain amount of the adsorbed oxygen group was adsorbed on the surface. At this time, the material has an initial resistance R_0_. When the sensitive material is placed in an oxidizing gas, the gas molecules adsorbed on the surface of the material will take away the free electrons in the semiconductor and become a negatively charged adsorbed molecule. At the same time, electron depletion layers are formed on the surface of the semiconductor. After the sensitive material loses carriers, its conductivity decreases. This resistance signal is measured and becomes the response of the sensitive material to gas. Since the surface has already chemically adsorbed the oxygen group that has captured the free electrons, when the sensitive material is placed in the reducing gas, the reducing gas molecules react with the chemically adsorbed oxygen, consuming the adsorbed oxygen and returning the free electrons to the sensitive material. The electron depletion layers are reduced. The number of carriers in the material increases, and the conductivity is also enhanced. Taking oxygen as the adsorbing molecules as an example, the chemical reaction that occurs on the surface of the material is as follows:

O_2_(gas)→O_2_(ads) (1)

O_2_(ads) + e^−^→O_2_^−^(ads) (2)

O_2_^−^(ads) + e^−^→2O^−^(ads) (3)

O^−^(ads) + e^−^→O^2−^(ads) (4)

Reactions (3) and (4) are carried out when the operating temperature is higher than 150 °C [31,32], and reaction (2) usually occurs when the temperature is lower than 150 °C [33].

The synthesized pure ZnO microspheres have a weak response to NO, which indicates that there is no effective traditional adsorption reaction on the surface of the material (Figure 5a), while the ZnO/CQDs composite has an enhanced NO response, which shows that doped CQDs have an important effect on gas sensitivity. The chemical properties of NO are very active, and this is because NO has an unpaired electron and it can easily form nitric oxide free radicals (•NO) [34]. It should be noted that although NO_2_ has the ability to accept electrons, nitrogen dioxide cannot spontaneously form •NO (nitrogen-oxygen free radicals). This spontaneous formation of free radicals is a unique characteristic of NO gas.

Many studies on carbon quantum dots have introduced the surface of carbon quantum dots with active functional groups, such as hydroxyl, amino, carboxyl and so on. Some of these functional groups were retained by incomplete carbonization of small-molecule organics, and some were modified to carbon quantum dots through surface activation reactions [34,35]. Since the doping and testing processes were conducted at a lower temperature (below 150 °C), many incompletely carbonized hydroxyl groups on the surface of CQDs are retained to the greatest extent, and react with nitric oxide radicals as follows [36,37]:

R-OH + •NO ⇋R-O• + HNO(ads)(5)

The generated intermediate product alcoxyl radical R-O• has an extremely high oxidation potential (2.8 eV) and has an intense tendency to capture electrons. In the ZnO/CQDs composite, the free electrons in the conduction band of ZnO could be captured.

R-O• + e^−^→R-O^−^ (6)

After the carriers’ concentration of ZnO was decreased, the conductivity of the material decreased, and the measured resistance value increased, which was displayed as a gas-sensitive response of the ZnO/CQDs composite. The composite material has little response to the oxidizing gas, which indicates that it is difficult for the oxidizing gas to directly deprive carriers from the composite material during the chemical adsorption process. The existence of carbon quantum dots transforms the gas adsorption reaction into a solid-phase contact reaction in the composite material, and electron transfer in the solid-phase contact reaction can more easily occur.

A major factor affecting the gas-sensing performance of traditional semiconductor gas-sensing materials is the morphology of the material. Materials with a porous micro-morphology have a large specific surface area, that is, a larger surface energy. The larger surface energy of the material tends to adsorb more oxygen molecules and test target gas molecules, and at this time, more surface gas-sensitive reactions will occur, resulting in greater gas-sensitive responses. Therefore, the preparation of porous materials with a large specific surface area has always been the focus of researchers. In the ZnO/CQDs composite, the material has a microspherical morphology of a lamellar assembly with a large specific surface area, which provides more contact with NO gas. At the same time, the countless active functional groups of the doped CQDs provide more gas-sensitive reaction sites with NO, increasing the gas sensitivity of the material.

## 4. Conclusions

In general, ZnO microspheres and CQDs were synthesized by a hydrothermal method, and CQDs were doped into ZnO by a grinding method to fabricate the ZnO/CQDs composite. Compared with pure ZnO microspheres, the ZnO/CQDs composite has a higher gas sensitivity response to NO gas. The ZnO/CQDs composite with the doped ratio of 100 mg ZnO doped with 10 uL CQDs (1 eq) achieved the best NO response. The response of the ZnO/CQDs composite to 100 ppm NO at an operating temperature of 100 °C was 238, the lowest detection limit was 100 ppb and the response and recovery times were 34 and 36 s, respectively. In addition, the sensitivity mechanism of the ZnO/CQDs composite to NO was also discussed and carbon quantum dots were determined to play an important role in NO gas sensitivity. The introduction of carbon quantum dots materials into ZnO materials may become a new method to improve the gas-sensing performance of sensitive materials, providing a new idea for NO sensing.
